# From warm to cold: migration of Adélie penguins within Cape Bird, Ross Island

**DOI:** 10.1038/srep11530

**Published:** 2015-06-26

**Authors:** Yaguang Nie, Liguang Sun, Xiaodong Liu, Steven D. Emslie

**Affiliations:** 1 Institute of Polar Environment, School of Earth and Space Sciences, University of Science and Technology of P. R. China, Hefei 230026, China; 2 Key Laboratory of Ion Beam Bioengineering, Hefei Institutes of Physical Science, Chinese Academy of Sciences and Anhui Province, Hefei, Anhui 230031, P. R. China; 3 Department of Biology and Marine Biology, University of North Carolina Wilmington, 601 S. College Road, Wilmington, NC 28403, USA

## Abstract

Due to their sensitivity to environmental change, penguins in Antarctica are widely used as bio-indicators in paleoclimatic research. On the basis of bio-element assemblages identified in four ornithogenic sediment profiles, we reconstructed the historical penguin population change at Cape Bird, Ross Island, for the past 1600 years. Clear succession of penguin population peaks were observed in different profiles at about 1400 AD, which suggested a high probability of migration within this region. The succession was most obviously marked by a sand layer lasting from 1400 to 1900 AD in one of the analyzed profiles. Multiple physical/chemical parameters indicated this sand layer was not formed in a lacustrine environment, but was marine-derived. Both isostatic subsidence and frequent storms under the colder climatic condition of the Little Ice Age were presumed to have caused the abandonment of the colonies, and we believe the penguins migrated from the coastal area of mid Cape Bird northward and to higher ground as recorded in the other sediment profiles. This migration was an ecological response to global climate change and possible subsequent geological effects in Antarctica.

Exploring climate change and corresponding ecological responses by species in the past are essential in understanding current and future trends of global warming and its impact on ecosystems^1^,^2^,^3^. Antarctica developed its unique ecosystem structures due to its isolation from the other continents and the absence of anthropogenic influence, making it an ideal location to study the natural interaction between climate and local ecosystems^4^,^5^. In this frozen land, the Adélie penguin (*Pygoscelis adeliae*) is one of the most well-known indicator species that is sensitive to climatic and environmental change^6^,^7^,^8^. Most previous research on the paleoecology of penguins has been based on biological remains excavated from abandoned colonies, which has provided important information on penguin occupation history, but is relatively weak in continuity over time^9^,^10^,^11^. As a bio-vector at a high trophic level, penguins play an important role in substance cycling between land and sea. They tend to enrich and transport marine-derived elements and nutrients to the terrestrial environment in the form of guano, eggshells, feathers, dropped food and carcasses, and have an important influence on the elemental composition of soil and sediments around their rookeries^12^,^13^,^14^, enabling us to explore their activities in a geochemical way. A bio-element assemblage in ornithogenic sediments is a set of elements that share the same origin from penguin guano. It provides an overall perspective from which to trace the source of multiple elements without being hindered by the specific geochemical behavior of an individual element, and potentiates the reconstruction of continuous records of penguin population change, which has been widely conducted in the ice-free areas around Antarctica^11^,^15^,^16^.

The Ross Sea is a high latitude embayment with a long history of Adélie penguin occupation^10^,^17^. More than 155,000 breeding pairs reside in the ice-free areas on Ross Island, forming one of the largest concentrations of this species in Antarctica^6^. However, research on ornithogenic sediments and the paleoecology of Adélie penguins on Ross Island have been limited in scope so far. A rise in penguin population inferred from one sediment core at Cape Bird during the cold period of the Little Ice Age (LIA) was reported^18^, and migration of Adélie penguins was among the possible scenarios behind this phenomenon. In this study, the physical/chemical proxies in four ornithogenic sediment profiles sampled from Cape Bird, Ross Island were investigated (Fig. 1 58,59). We compared the historical penguin population change inferred from bio-element assemblages dating to the past 1600 years and discuss possible reasons for the spatial change of occupation patterns observed. On the basis of multiple geophysical and geochemical parameters measured in one of the profiles (MB4 on Fig. 1), the sand layer between 15 and 5 cm was deemed to be marine-derived and provided insights into possible ecological responses to global climate change and subsequent geological processes in Antarctica.

## Results

To extract information on the paleoecology of Adélie penguins stored in the sediment cores encoded MB4, CL2, MB1 and MB6, we employed bio-elements assemblages which have been successfully used in related research as a proxy for penguin population change^11^,^15^. In our previous geochemical study on the ornithogenic profiles from the Ross Sea region including MB4, MB6 and CL2, As, Cd, Cu, P, S, Se and Zn were identified as the penguin bio-element assemblage in this region through analysis on end-member environmental media and multiple statistical methods^14^, indicating their common source. Additionally, F and Hg were also found to be enriched in penguin guano, sharing the same change patterns in the profiles with the other bio-elements^19^,^20^. As an additional test, we measured Al, As, Ba, Ca, Co, Cr, Cu, F, Fe, Hg, K, Mg, Mn, Na, Ni, P, S, Se, Sr, Ti and Zn in MB1, and conducted cluster analysis on these elements (see Figs 2 in Appendix). As, Cu, F, Hg, S, Se, and Zn were found to cluster in the same group with the typical bio-element P, which is highly concentrated in penguin guano^14^,^21^,^22^, forming the bio-element assemblage for profile MB1. This result agrees well with the other three profiles except that Cd was not measured in MB1, showing a regional geochemical consistency.

To determine the primary controlling factor for this assemblage, we performed Q factor analysis (QFA) on the bio-elements in the different profiles, and found that more than 70% of the variance in the data could be explained by the first two factors (Table 1). Factor 1, which accounts for at least more than 50% of the total variance, resembled the pattern of bio-elements with depth in the four profiles, and thus was deemed to represent the relative abundance of guano input in the sediments over time. Plotting the loadings for Factor 1 of the profiles with age sequences, we drafted the penguin population change of different sites within Cape Bird dating to the past 1600 years (Fig. 2b). As can be seen, profile MB4 has the longest time span, which dates from about 390 AD. The penguin population was rather stable at the beginning, and began to increase at about 900 AD. It reached its peak population size between 900 and 1400 AD, and then dropped to a very low level, lasting for about 500 years. At around 1900 AD, the population increased again to the level before 900 AD. Profile CL2 (~605 AD) is slightly younger than MB4, and it showed a peak in penguin population between 950 and 1400 AD, corresponding to that in MB4. A population decline was also observed at 1400 AD in CL2, coinciding with the decrease of penguin population documented in MB4, and the population remained low with some fluctuations to the present. The time span of MB1 (~574 AD) is similar to CL2, indicating a decreasing population trend from the oldest layers to about 1300 AD with a sudden peak between 1300 and 1500 AD. After that, the penguin population dropped to the level before 1300 AD. MB6 (~1280 AD) is not as deep as the other profiles, spanning a period of more than 600 years. The curve for factor loading in MB6 rose to a high level at 1400 AD and lasted about 250 years, then reduced to a low level until a slight increase in modern times. In comparison, only MB4 and CL2 showed penguin population peaks at similar time spans, probably due to their adjoining locations. Penguin population peaks in MB6 and MB1 do not agree well with MB4 and CL2, but display a clear phase difference. These two sites seem to become more populated right after the decrease of penguins in mid Cape Bird at around 1400 AD.

The key point of the penguin population change reconstructed above lies in a critical time span between 1400 and 1900 AD when MB4 and CL2 showed a sharp decline. As documented in the field records, the sediments in MB4 below 15 cm (before ~1400 AD) mainly consisted of fine-grained ornithogenic sediments with a rancid smell (typical for a young and developing soil); the sediment unit between 15 and 5 cm (from 1400 to 1900 AD, corresponding to the critical time span) was dominated by dark-colored coarse sands; the surface layer above 5 cm (after 1900 AD) was rich in fine black algal residues^19^. Since the sand layer was unique and never observed in other profiles, we conducted specific physical and chemical analyses including grain size, magnetic susceptibility (MS), total organic carbon (TOC), total nitrogen (TN), Sr/Ba (on fine sediments) and calculated TOC/TN on MB4 (Fig. 3) to investigate the possible cause for the formation of the sand layer. Results for grain size analysis show that fine clay (<2 μm) is minor in MB4, only accounting for about 0.5% of the bulk sediment. The distribution of clay is constant with depth compared to the apparent shift of lithology through the profile. Silt (2 ~ 63 μm) comprises a larger percentage of sediment at 12.01%, while sand (63 μm~2 mm) acts as the major component of the sediments (about 87.94%). Silt remained low and fluctuating before 900 AD, and reached its peak (about 30%) at around 1300 AD. A sudden trough occurred between 1400 and 1900 AD, and the ratio of silt in the sediments raised again in the most recent 100 years. Due to the low content of clay, sand showed an opposite trend to silt. Mean grain size (Mz) increased gradually from the bottom but dropped to low values (corresponding to coarser grain size) between 1400 and 1900 AD, while MS elevated in this layer. We also compared grain size distribution in several samples from different depths in MB4 (Fig. 4). It is apparent that the layers above 5 cm and below 15 cm (e.g. MB4-3, MB4-5, MB4-25, MB4-35, MB4-45) are characterized by a wide grain-size distribution range with multiple peaks. In contrast, the sand layer samples (MB4-11, MB4-13, MB4-16, MB4-17) showed sharp mono-peaks with very narrow grain-size distribution range on the high end diameter axis. TOC and TN were both low before 900 AD followed by a peak at around 1300 AD. After a trough in the sand layer, TOC and TN bounced back to a high level in the surface of the profile (Fig. 3). Sr/Ba in the fine sediment ranged from ~1.5 to 4.5, but was apparently lower in the sand layer. TOC/TN in the sand layer was much higher (>15) in the remaining section (<10).

## Discussion

### Change in the sedimentary environment recorded in MB4

The succession of penguin population peaks along the same time sequence in different sites of the same region rendered a high probability of migration within the region. As illustrated in Fig. 2a^59^, it’s very likely that penguins abandoned their colonies in the coastal area of mid Cape Bird (MB4 and CL2) around 1400 AD, and moved northward (MB6) and to higher ground (MB1). As stated above, our attentions were drawn to one particular profile whose unique sand layer occurred just after the emigration of penguins from mid Cape Bird. To verify the hypothesis of migration deduced from penguin population reconstruction, we conducted several physical and chemical analyses to explore the possible environmental change that might lead to the abandonment of penguin colonies at mid Cape Bird.

Previous study on lithology and material source of this profile^14^,^19^ indicated that MB4 received influence from penguin guano and endogenous freshwater algae, and the two constituents comprised most of the OM in the sediments. Since algae biomass is greatly influenced by guano input as its nutrient supply^23^,^24^, the similar pattern of TOC and TN (as indicator for OM, Fig. 3) with bio-elements and pigments (Fig. 4 in Appendix) is understandable. Thus the troughs observed in TOC and TN between 1400 and 1900 AD were indicative of a period with reduced penguin population and algae in the lake at this site. Silt was also found to share a similar pattern over time with TOC and TN, suggesting most OM was bound to sediments at this grain size. Sand, on the other hand, showed an opposite trend to that of OM and silt, showing the dominance of inorganic mineral at this grain size. High MS and low Mz in the sand layer was attributed to the increased level of inorganic mineral input corroborated by high Al, Co, Cr, Fe, Ni, Si and Ti contents (see Fig. 4 in Appendix). According to the analyses on grain size distribution of different depth of the sediments, it is evident that the sand layer samples are homogeneous with a uniform origin of materials and were not subjected to resizing after deposition, while the remaining parts are from multiple mixed sources. In addition, we calculated mean grain size parameters in both ornithogenic and sand layers (Table 2). Except that standard deviation shows poor gradation in both layers, skewness and kurtosis were in accordance with grain size parameters and distribution (Figs 3,4). Thus, on the basis of lithology and the results from TOC, TN, grain size and magnetic susceptibility, the sand layer corresponded to coarser sediments with little biological influence, which was distinctively different from the rest of the profile. There was an apparent shift in material source in this layer, possibly caused by an incident of environmental change with massive exogenous material input in the pond. Considering the location of the sampling site of MB4, the coarse material could either be marine or glacial in origin.

We employed Sr/Ba in the fine sediments and TOC/TN in the bulk sediments to determine the origin of this specific sand layer. Samples from different depths in MB4 were processed to extract fine sediments for Sr and Ba analysis (Fig. 3), since the constituent at smaller grain size is better for source tracing^25^. Sr and Ba are both alkali metals with similar chemical properties, but they fractionate under different depositional environments so that Sr/Ba is applicable to determine the source of the sediments. Normally, marine-derived sediments have higher Sr/Ba and a ratio higher than 1 indicates a marine-origin. Thus, our results with Sr/Ba > 1 in the sand layer demonstrates a clear marine influence. However, Sr/Ba in the ornithogenic sediments which were formed under a lacustrine environment (indicated by pigments from freshwater algae) is even higher. This higher ratio results from greatly enriched Sr in the ornithogenic sediments with the continuous input of penguin guano, which also has a strong marine signature^11^,^15^. Like Sr/Ba, TOC/TN in the ornithogenic sediments is related to a clear marine influence (TOC/TN < 12^26^) with low values (9.19, n = 40, Fig. 3), in accordance with TOC/TN measured in fresh guano (2.72, n = 3) and algae (7.51, n = 6). TOC/TN in the sand layer was generally higher (14.17, n = 13), indicating a greater decrease in TN than TOC since the nitrogen pool is expected to be more sensitive to the absence of ornithogenic influence^27^. According to Hodgson’s reaearch^28^, TOC/TN and δ^13^C (−21.48‰, n = 7, Liu *et al.*, 2013) in the sand layer of MB4 lie in the range of marine DOC. Given that guano was not present in this layer, both Sr/Ba in the fine sediments and TOC/TN-δ^13^C in the bulk sediments provide strong evidence for its origin from the sea instead of inland. Most important, if the sand layer was caused by advancing glaciers, the penguin population peak in MB1 wouldn’t appear after MB4 as we observed. Moreover, there is no clear evidence of massive glacier advance recorded in the Ross Sea region during the time span^29^. Thus, both the analysis on the origin of the sand layer in MB4 and the result of penguin population reconstruction indicated the layer was marine-derived.

### Possible scenarios for the formation of sand layer induced by climate changes

Our results indicate that the sand layer in MB4 records a major environmental change that caused a massive input of marine-derived sediments from 1400 to 1900 AD. This period is in accordance with that of the Little Ice Age (LIA), the latest and the most prominent climate change event in the past 2000 years^30^. Originally defined as a fluctuating cool climate in the Northern Hemisphere marked by glacier advances in Europe from the 15^th^ to 19^th^ centuries^31^, the LIA is now considered to have been a global phenomenon with more and more supportive evidence^32^. Multiple records in Antarctica provide evidence for LIA impacts in this southernmost continent, though the actual duration of the LIA differs among regions^33^,^34^,^35^. In the most recently published report from PAGES 2 k Consortium^36^, synthetic temperature reconstructions using different data sources exhibit a clear cooling trend during the LIA in Antarctica. As for the Ross Sea region, research on ice cores also indicates a lower temperature during that period. According to a study on δD of an ice core from Mt. Erebus Saddle (located on Ross Island), the Ross Sea region was 1.6 ± 1.4 °C colder in the LIA than the post-LIA period^32^. Similarly, Bertler *et al.*^37^ reported their isotopic analysis on an ice core retrieved from the Victoria Lower Glacier, showing the surface temperatures in the Ross Sea region were about 2 °C colder during the LIA with stronger katabatic flow and probably increased sea ice.

Considering these records, we investigated the connection between climate change in the Ross Sea during the LIA and the appearance of the sand layer in MB4. Two possible scenarios could explain the phenomenon. The first one involves isostatic effects and the cold conditions. As is well known, the evolution of climate has substantial influence on the ice mass in Antarctica, resulting in isostatic movements and fluctuation in relative sea level^38^. In the Holocene, massive ice accumulation during the Last Glacial Maximum (LGM) melted due to the warming climate, in turn leading to a subsequent isostatic uplift of the continent. Coastal areas are usually more sensitive and susceptible to isostatic effects^39^. For example, Late Quaternary marine deposits were found at elevations as high as 60 m above sea level along the Victoria Land coastline and on the lower flanks of late Cenozoic volcanic islands of McMurdo Sound^40^. According to Dochat *et al.*^41^, the low-elevation beach ridges at Cape Bird were most likely formed as a result of isostatic uplift since seasonally open water appeared off the coast following recession of an ice-shelf front at about 3585 ^14^C a BP. We believe that a cooler climate during the LIA in the Ross Sea region could have caused a reverse effect: an increase of ice accumulation on Ross Island and subsequent isostatic subsidence. If so, the site of MB4 would receive marine-derived sediments, first possibly as a lagoon, causing deposition of marine-derived sediments observed as the sand layer. However, profile CL2, which was excavated from a site very close to MB4 (but more inland-ward) lacked a similar sand layer, though its bio-element assemblage did indicate a period with decreasing penguin populations corresponding to the sand layer in MB4. Thus, it’s quite likely that the boundary of inland-ward movement of the shoreline lay just between the sites of MB4 and CL2 during 1400 and 1900 AD (Fig. 5 41). After 1900 AD, the beach ridges rose again to their current status due to the warming climate in recent centuries.

The second scenario suggests that the sand layer was likely brought by storm surges during the LIA under more severe climate conditions. Storms are highly energetic with stunning transporting capacity, which may impose heavy influence on costal area sediments^42^,^43^. During storms surges, sea water may invade the low altitude beach ridges with a range of kilometers, leaving sand layers and stratigraphic sequences as has been observed in MB4^44^,^45^. Considering the relationship between storms and barometric pressure, the Ross Sea region may have experienced severe storms with the conjunction of three different air masses from Victoria Land, the Ross Sea, and the Ross Ice Shelf^46^,^47^. It has been documented that during the LIA, though large spatial and temporal variability existed among regions, Antarctica was cooler and stormier overall^33^,^37^,^48^. Under such circumstances, the lacustrine environment in the sampling site of MB4 would be altered by the continuous storms (change of water chemical properties, massive input of marine-derived sediments, and landform reshaped by the waves), which in turn caused the abandonment of the colonies and the demise of the freshwater algae as well. Thus we believe, apart from the possibility of isostatic subsidence, more frequent storm surges could also result in a marine-derived sand layer on top of lacustrine ornithogenic sediments, though both scenarios were induced by the climate changes during the LIA. At the same time, the presence of these two processes induced by colder LIA climate conditions could cause negative effects on the population of Adélie penguins inhabiting the MB4 site, and this might lead to the migration of penguins to other suitable nesting sites.

### Penguin ecological response to environmental changes

Given the fact that the Ross Sea region is biologically productive with a long history of penguin occupation^49^, such drastic environmental changes would surely lead to disturbance in the local ecology. Investigations of abandoned colonies in this region indicate that penguin populations experienced a sudden decrease after a long period of expansion of colonies into the southern Ross Sea between 4000 and 2000 a BP, a period known as “penguin optimum”^10^,^17^. By ~2000 a BP, a cooler period led to an increase in sea ice that blocked access by penguins to the Scott Coast, causing abandonment of the southern Ross Sea by breeding penguins that remains unchanged today^9^,^10^. It also has been hypothesized that the period between 2300 and 1100 BP was warmer, leading to an expansion of southern elephant seals (*Mirounga leonina*) into the Ross Sea Embayment which inhibited the presence and settling of Adélie penguins^50^. However, no *bona fide* evidence (fetal remains, pup bones) of elephant seals has been located in the Ross Sea, and seal hair deposits and adult bones that have been reported by Hall *et al.*^50^ were more likely from molting animals migrating into the Ross Sea at various times in the past. Penguin reoccupation of the southern Ross Sea commenced from the 8^th^ to 14^th^ centuries^10^,^17^.

Our data on penguin population reconstruction in MB4 and CL2 are consistent with these records, showing an increase from 900 AD and the abandonment of the colonies after 1400 AD. We assume that the LIA was accompanied by relatively rapid climatic and environmental changes in the Ross Sea region, and the impacted ice-free areas at Cape Bird that are crucial for breeding penguins. With the inland-ward movement of the shoreline, the colonies nearest the beach would have been submerged, or the frequent storms deteriorated the conditions in the colonies, making them unfit for the penguins. Furthermore, expanding sea ice would have blocked beach access in many locations, rendering the breeding sites difficult to reach^51^,^52^. Under such circumstances, the penguins originally inhabiting mid Cape Bird would decrease, possibly moving northward (as indicated at MB6) and to higher ground (as indicated at MB1). At the same time, with a complete change in the aquatic environment and the absence of nutrient supply from penguin guano, algae in the pond died out quickly when the coarse sands poured in as indicated by the pigments. After 1900 AD when the site once again become lacustrine, algae reappeared with a small number of penguins along the coastal area of mid Cape Bird (Fig. 5).

## Methods

### Sampling

**Figure 1:**
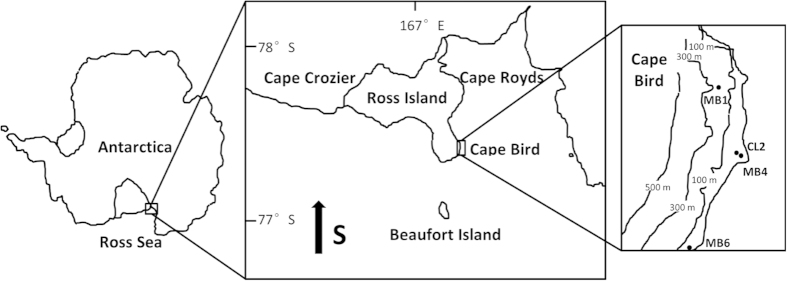
58,59 Research area and sampling sites on Ross Island and Cape Bird, Ross Sea, Antarctica.

**Figure 2:**
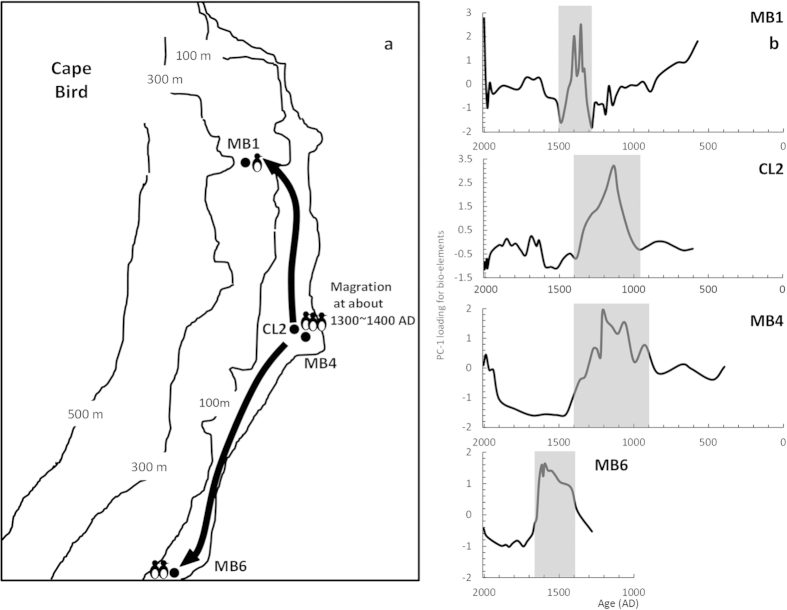
59 Penguin population change inferred from four profiles from Cape Bird and a possible migration route during 1300 ~ 1400 AD.

The sediment profiles in this study were collected in McMurdo Sound, the Ross Sea region, in January 2010 (Fig. 1). The excavation sites were located at Cape Bird (~15 km^2^). Profile MB4 (42 cm) was taken in a small pond between the fourth and fifth beach ridges on the coast of mid Cape Bird (166°22′25.6″E, 77°14′35.3″S). A larger adjoining pond is located on the fifth beach ridge above the sea level, and CL2 (35 cm) was excavated on its northern edge (166° 22′ 25.6′′ E, 77° 14′ 35.3′′ S). Abandoned penguin colonies were found near their sampling sites, indicating possible ornithogenic influence in the pond from penguin guano. Profile MB1 (51 cm) was collected from a dried-out catchment on an elevated hill side south to the sampling sites of MB4 and CL2 (166° 22′ 26′′ E, 77° 14′ 35.7′′ S) with clear traces of penguin activities. The MB6 profile (38 cm) was also taken from a dried-out catchment on the second terrace above sea level at an active penguin colony in northern Cape Bird (166˚ 26′ 44.4′′ E, 77˚12′ 47.5′′ S). Details of sectioning and lithology of the profiles were documented in Nie *et al.*^19^. All the samples were kept at −20 ˚C in the laboratory prior to chemical analysis. They were air-dried and homogenized by grinding after the careful removal of large rock fragments and biological remains. Samples for pigment analysis were separated and freeze-dried. The final powder samples were passed through a 74 μm mesh sieve.

### Physical/chemical analysis

**Figure 3:**
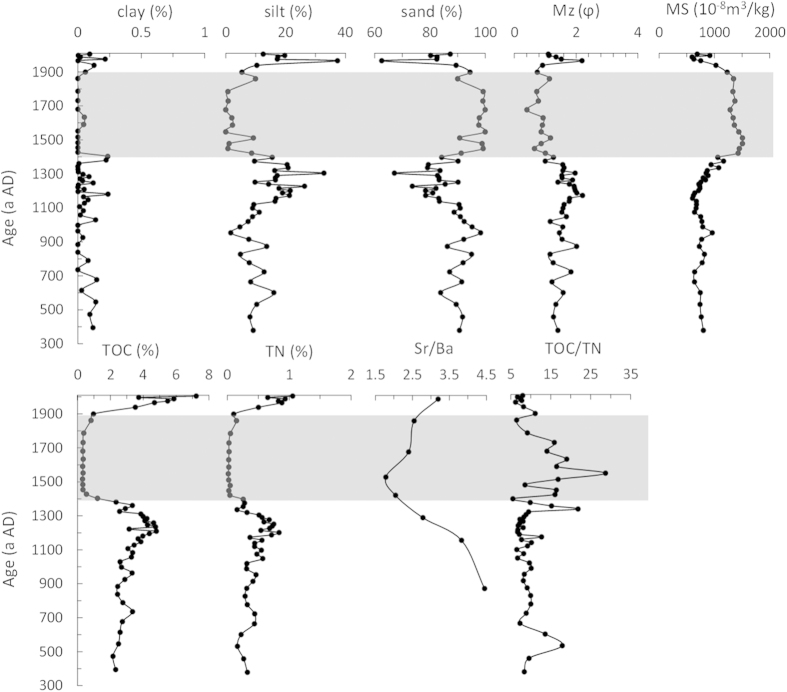
Grain size parameters, MS, TOC, TN, Sr/Ba (in fine sediments) and TOC/TN in profile MB4 by age, with shaded area representing the time span of the sand layer (1400 - 1900 AD).

**Figure 4:**
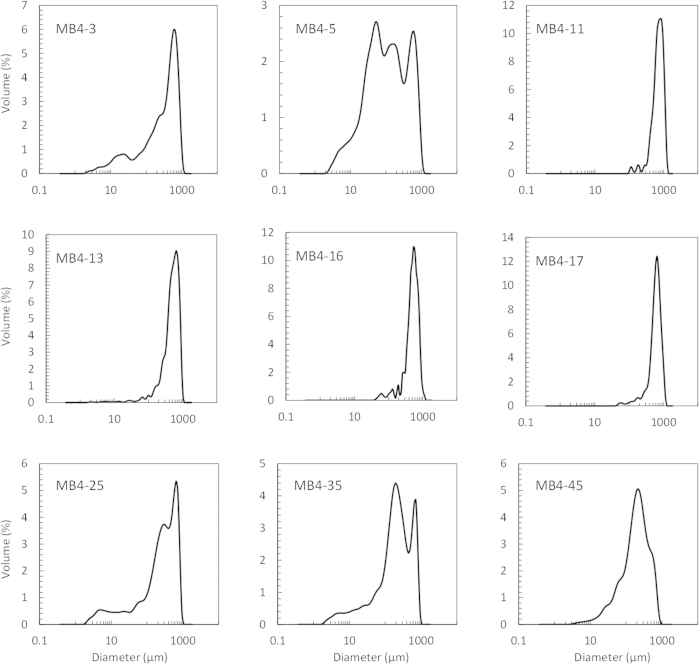
Grain size distribution of selected subsamples from the sand and ornithogenic layers.

**Figure 5:**
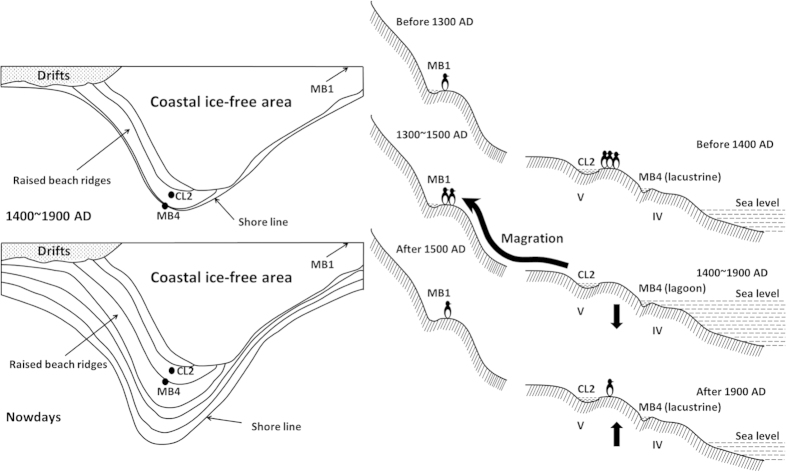
41 Isostatic movement in the coastal area of mid Cape Bird and subsequent ecological response by penguins.

For grain size analysis of profile MB4, 0.1 g air-dried sample before grinding was heated to 150 ˚C with 10 ~ 20 mL H_2_O_2_ for 30 min, then 10 mL HCl (10%) was added with heating to 80 ˚C until no air bubbles were observed. After the removal of the supernatant following centrifugation, 10 mL deionized water was added and centrifugation was repeated. The samples were analyzed on a Laser Particle Size Analyzer (LS 13320) after 10 mL sodium hexametaphosphate (as dispersing agent) was added to the centrifuge tubes. Magnetic susceptibility (MS) of the MB4 samples was measured on air-dried samples before grinding by a susceptibility meter (MS2, Bartington). Total nitrogen (TN) and sulfur (S) in MB4 and S in MB1 were determined by an elemental analyzer (vario EL III) with a relative standard deviation (RSD) less than 1%. Chemical volumetric method was employed to measure total organic carbon (TOC) in MB4. Al, Ca, Fe, K, Na and Si in MB4 were measured on an X-ray fluorescence analyzer (XRF-1800, Shimadzu) in the Instruments’ Center for Physical Science of University of Science and Technology of China with an RSD less than 0.5%. As, Hg and Se in both MB4 and MB1 were determined by Atomic Fluorescence Spectrometry (AFS-930, Titan Instruments Co. Ltd.). Cd, Co, Cr, Cu, Mn, Ni, P, and Ti in MB4 and Al, Ba, Ca, Co, Cr, Cu, Fe, K, Mg, Mn, Na, Ni, P, Sr, Ti and Zn in MB1 were measured by inductively coupled plasma-optical emission spectroscopy (ICP-OES, Perkin Elmer 2100DV). Detailed information on the elements measured above were presented in Liu *et al.*^14^ and Nie *et al.*^19^. Fluorine (F) in both MB4 and MB1 was determined by ion selective electrode as described in Nie *et al.*^20^. Sr and Ba in the fine sediments of MB4 were also measured by ICP-OES. The fine sediment samples were obtained by directly passing the dried sediments through a 74 μm mesh sieve without grinding. Due to the low content of fine sediments in the sand layer of MB4, they were measured on mixed samples and plotted against mean depth (Fig. 3). Pigments including chlorophyll a (Chl a), chlorophyll b (Chl b), canthaxanthin (Can), echinenone (Ech), β-carotene (β-Car) and α-carotene (α-Car) in profile MB4 were analyzed using high performance liquid chromatography (HPLC, Finnigan Surveyor, Thermo Electron, USA) coupled with triple quadrupole mass spectrometer (Finnigan TSQ Quantum Discovery MAX, Thermo Electron, USA). Samples were pretreated following the steps from the research of Leavitt and Hodgson^53^ and Leeben^54^. Pigments were extracted from freeze-dried sediment samples, purified by filtration, diluted to a constant volume with methanol and then measured. Detailed methodology with chromatographic conditions and mass spectrometer settings can be found in Chen *et al.*^55^.

### Grain size parameter calculation

The parameters were calculated according to Folk and Ward^56^ with the formula listed below.


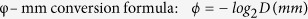



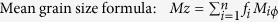



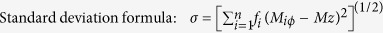



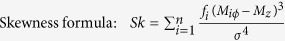



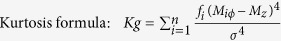
Where: M_iφ_ = channel center; fi = the percentage of particles in i’th channel.

### Dating

Chronology of the profiles was determined by ^210^Pb dating and AMS ^14^C dating. For ^210^Pb dating, the radioactivity of the samples in the upper layers of the profiles was measured by direct gamma spectrometry using Ortec HPGe GWL series, well-type, coaxial, low background, intrinsic germanium detector. The samples were first dried to constant weight at 105 °C, then packed into centrifuge tubes for a standing of three weeks so that radioactive equilibration could be reached before analysis. Spectra were obtained for a count time about 24 h to gain enough counts. According to the spectrum of standard radionuclide samples, ^210^Pb, ^226^Ra (activity was determined by gamma-ray emissions of its daughter isotope ^214^Pb) and ^137^Cs were measured at 46.5 keV, 295 keV and 662 keV, respectively (Fig. 1 in Appendix. More methodological details about ^210^Pb dating can be found in Xu *et al.*^57^). AMS ^14^C dating was performed on bulk sediments and biological remains picked from the profiles by Keck Carbon Cycle AMS Facility, University of California, Irvine. Conventional dates from penguin remains were calibrated using a ΔR = 750 ± 50 (Emslie *et al.*^10^) for the marine carbon reservoir effect in this region using CALIB 5.0 software program with the INTCAL98 calibration dataset. The bulk sediment in the bottom layer of MB4 was considered under minor penguin influence, and the date from it was calibrated to atmosphere dataset. AMS ^14^C dating results with calibrated age and ranges are provided in the Appendix (in Table 1), as well as detailed discussion on dating of the profiles.

## Additional Information

**How to cite this article**: Nie, Y. *et al.* From warm to cold: migration of Adélie penguins within Cape Bird, Ross Island. *Sci. Rep.* **5**, 11530; doi: 10.1038/srep11530 (2015).

## Supplementary Material

Supplementary Appendix

## Figures and Tables

**Table 1:** Bio-elements used for QFA in the profiles and variance explained by Factor 1 and 2.

profile	bio-elements used in QFA	% of variance for Factor 1	% of variance for Factor 2	cumulative %
MB4	Cu, Cd, P, As, Se, S, Hg and F	69.753	10.237	79.99
MB6	Cu, Zn, Cd, P, As, Se, S, Hg and F	78.906	8.967	87.873
CL2	Cu, Zn, Cd, P, Se, S, Hg and F	66.624	15.685	82.309
MB1	Cu, Zn, P, As, Se, S, Hg and F	53.66	18.582	72.242

**Table 2:** Mean grain size parameters of layers with different lithology in MB4.

Layer	n	Mz/φ	σ/φ	Sk	Kg
Ornithogeinc	40	1.57	1.94	0.84	3.02
Sand	13	0.88	1.97	0.15	2.64
