# Supplementary material for: From warm to cold: migration of Adélie penguins within Cape Bird, Ross Island
Source: Sci Rep. 2015 Jun 26;5:11530. doi: 10.1038/srep11530 (PMC4650636; doi:10.1038/srep11530)
Supplement: Supplementary Appendix [file srep11530-s1.doc]

Appendix for the manuscript:

**From warm to cold: migration of Adélie penguins within Cape Bird, Ross Island**

**Yaguang Nie^1, 2^*, Liguang Sun^1^*, Xiaodong Liu^1^*, Steven D. Emslie^3^**

**^1^Institute of Polar Environment, School of Earth and Space Sciences, University of Science and Technology of China, Hefei 230026, China; ^2^Key Laboratory of Ion Beam Bioengineering, Hefei Institutes of Physical Science, Chinese Academy of Sciences and Anhui Province, Hefei, Anhui 230031, P. R. China; ^3^Department of Biology and Marine Biology, University of North Carolina Wilmington, 601 S. College Road, Wilmington, NC 28403, USA.**

**Correspondence and requests for materials should be addressed to L.-G.S. (slg@ustc.edu.cn) or X.-D.L. (ycx@ustc.edu.cn); * these authors contributed equally to this work**

**Detailed discussion on dating.** Due to the pristine environment in the Ross Sea region and the lack of human activities, we believe no disturbance had come to the profiles. ^210^Pb dating on the samples from the upper layers were performed, and produced enough data for age calculation. As shown in Fig. 1a, activity of ^210^Pb in MB4 displayed a down trend with fluctuations against depth, while that of ^226^Ra was increasing gradually. The two radionuclides came to equilibration at 8 cm, indicating the decreasing excess ^210^Pb (^210^Pb_ex_) reached its zero point. Activity of ^210^Pb and ^226^Ra in MB6 shared the same pattern down the depth core with a sudden peak at 2.5 cm, and the zero point was reached at 5.5 cm. ^210^Pb and ^226^Ra in CL2 almost came to equilibration at 3.5 cm, then ^210^Pb_ex_ raised until eventually returned to the zero point at 7 cm. Activity of ^210^Pb in MB1 kept decreasing except a rise between 3 and 5 cm with zero point being reached at 9 cm. Since ^210^Pb in the measured profiles did not decline with depth in an exponential way, probably attributed to the changes in sedimentation rate, a Constant Rate of Supply (CRS) model was used for dating calculation^1^. The result showed that at the depth of 6.4 cm, 4.2 cm, 6 cm and 8 cm in MB4, MB6, Cl2 and MB1, the profiles dated back to 1861, 1891, 1906 and 1875 AD, respectively. ^137^Cs is a nuclear testing-produced radionuclide originated mainly from the northern hemisphere, and its signal was generally weak after deposition due to long distance transportation in the atmosphere^2^. Fig. 1a showed a low level of ^137^Cs activity in MB4, MB6 and CL2 (several Bq/kg), which is consistent with that of Antarctic sediments^3, 4^. The ^137^Cs peak in MB4 was rather apparent at 1988, and might be corresponding to the Chernobyl nuclear accident in 1986, while ^137^Cs peaks in MB6, CL2, and MB1 were presented in the surface layer with no signature for the bomb testing in 60s and the accident in 1986. The high ^137^Cs activity in the surface of MB1 was much higher than the other profiles, probably caused by the wash-in from the catchment, for this site is closer to the front of glaciers.

^14^C age at different depths were obtained through AMS ^14^C dating in the four profiles (Table 1). The bulk sediment sample in MB4 has a mixed source in total carbon, and was believed to receive minor influence from penguin guano. Thus, the date was calibrated to atmosphere dataset to better fit the age sequence of the profile. The rest of the dates from biological remains and ornithogenic sediments were considered under clear influence of marine reservoir effect. On the basis of the investigations made on ^14^C age of modern penguin bones, a ΔR=750±50 would be practicable for the Ross Sea region^5^, and these dates were calibrated to marine dataset accordingly. Combining ^210^Pb dates and with ^14^C dates, we used four polynomial curves fitted to describe age-depth relationship in each profile (Fig. 1b). As can be seen in the figure, profile MB4, MB6, CL2 and MB1 dated back to 394, 1281, 605 and 574 AD.

**References**

1. Appleby, P. (2001). Chronostratigraphic techniques in recent sediments Tracking environmental change using lake sediments (pp. 171-203): Springer.

2. Appleby, P. (2008). Three decades of dating recent sediments by fallout radionuclides: a review. The Holocene, 18(1), 83-93.

3. Sanders, C. J., Santos, I. R., Patchineelam, S. R., Schaefer, C., Silva-Filho, E. V. (2010). Recent 137Cs deposition in sediments of Admiralty Bay, Antarctica. Journal of environmental radioactivity, 101(5), 421-424.

4. Ferreira, P. A. d. L., Ribeiro, A. P., Nascimento, M. G. d., Martins, C. d. C., Mahiques, M. M. d., Montone, R. C., Figueira, R. C. L. (2013). 137Cs in marine sediments of Admiralty Bay, King George Island, Antarctica. Science of the Total Environment, 443, 505-510.

5. Emslie, S. D., Coats, L., Licht, K. (2007). A 45,000 yr record of Adélie penguins and climate change in the Ross Sea, Antarctica. Geology, 35(1), 61-64.

Table 1 Results for AMS ^14^C dating in profile MB4

| Depth (cm) | Material | Conventional ^14^C age (BP) | ^14^C age clbi (AD) | |
| --- | --- | --- | --- | --- |
| MB4-17 | bone | 1775 ± 15 | 1281~1433 | 1334 |
| MB4-20 | bone | 1790 ± 15 | 1269~1425 | 1325 |
| MB4-25 | feather | 1965 ± 15 | 1063~1293 | 1210 |
| MB4-36 | bulk sediment | 1180 ± 35 | 728~975 | 885 |
|  |  |  |  |  |
| MB6-24 | bulk sediment | 1445 ±15 | 1498~1691 | 1638 |
| MB6-28.8 | bulk sediment | 1475 ± 20 | 1476~1679 | 1574 |
| MB5-30.5 | bone | 1525 ± 15 | 1449~1652 | 1520 |
| MB6-37.8 | hair | 1880 ± 15 | 1184~1334 | 1283 |
|  |  |  |  |  |
| CL2-14.5 | bulk sediment | 1290 ± 60 | 1637~1799 | 1637 |
| CL2-22.5 | bulk sediment | 2005 ± 30 | 1030~1280 | 1280 |
| CL2-26 | bulk sediment | 2125 ± 30 | 907~1164 | 1029 |
| CL2-30.5 | bulk sediment | 2235 ± 45 | 773~1043 | 2122 |
|  |  |  |  |  |
| MB1-12.5 | bone | 1560 ± 15 | 1433~1630 | 1492 |
| MB1-16.5 | bone | 1605 ± 15 | 1409~1536 | 1463 |
| MB1-20.5 | bone | 1620 ± 15 | 1401~1526 | 1455 |
| MB1-30 | bone | 1795 ± 15 | 1264~1422 | 1323 |
| MB1-40 | bone | 2065 ± 15 | 998~1215 | 1067 |
| MB1-50.5 | bone | 2540 ± 15 | 519~700 | 633 |

Figure-1 Activity of radionuclides measured in the ornithogenic sediment profiles from Cape Bird and the result of ^210^Pb dating

Figure-2 Vertical distributions versus depth of analyzed elements including Al, As, Ba, Ca, Co, Cr, Cu, Fe, K, Mg, Mn, Na, Ni, P, S, Se, Sr, Ti, Zn and F in profile MB1

Figure-3 Clustering results for measured elements in profile MB1

Figure-4 Lithological elements (line 1), bio-elements (line 2 and 3) and pigments (line 4) in profile MB4 against age
